# A Low-Cost, Low-Power, Multisensory Device and Multivariable Time Series Prediction for Beehive Health Monitoring

**DOI:** 10.3390/s23031407

**Published:** 2023-01-27

**Authors:** Iraklis Rigakis, Ilyas Potamitis, Nicolas-Alexander Tatlas, Giota Psirofonia, Efsevia Tzagaraki, Eleftherios Alissandrakis

**Affiliations:** 1 INSECTRONICS, 55 An. Mantaka Str, Chania, GR-73100 Crete, Greece; 2 Department of Electrical and Electronics Engineering, University of West Attica, 12244 Athens, Greece; 3 Department of Music Technology & Acoustics, Hellenic Mediterranean University, 74100 Rethymno, Greece; 4 Department of Agriculture, Hellenic Mediterranean University, 71410 Heraklion, Greece

**Keywords:** apis mellifera, beehive monitoring, remote sensing, time series prediction

## Abstract

We present a custom platform that integrates data from several sensors measuring synchronously different variables of the beehive and wirelessly transmits all measurements to a cloud server. There is a rich literature on beehive monitoring. The choice of our work is not to use ready platforms such as Arduino and Raspberry Pi and to present a low cost and power solution for long term monitoring. We integrate sensors that are not limited to the typical toolbox of beehive monitoring such as gas, vibrations and bee counters. The synchronous sampling of all sensors every 5 min allows us to form a multivariable time series that serves in two ways: (a) it provides immediate alerting in case a measurement exceeds predefined boundaries that are known to characterize a healthy beehive, and (b) based on historical data predict future levels that are correlated with hive’s health. Finally, we demonstrate the benefit of using additional regressors in the prediction of the variables of interest. The database, the code and a video of the vibrational activity of two months are made open to the interested readers.

## 1. Introduction

Beekeepers in Europe, America and Asia have been reporting weakening bee numbers and colony losses. There are repeated reports that bee colonies are declining every year [1]. Only regarding the case of honeybees (*Apis mellifera*), the number of beekeepers in the EU is estimated to be approximately 700,000, keeping more than 15 million hives, and honey production is estimated to be close to 280,000 tons/year [2]. *Apis mellifera* is the only managed honeybee species in Europe providing food, which has been domesticated by beekeepers to produce beekeeping products (i.e. honey, pollen, venom, wax, etc.). However, the most important role of honeybees is not honey production but the pollination of many agricultural crops. In numbers, the direct value of honey produced in the EU is estimated about EUR 140 million, while the value of insect pollination for European agriculture has been estimated to be around EUR 20 billion per year, and EUR 153 billion worldwide [3]. When wild bees do not visit or are not enough to pollinate agricultural fields, managed honeybee hives are often the only solution for farmers to ensure crop pollination. Pollinators decline results in loss of pollination services which have negative ecological and economic impacts significantly affecting the maintenance of wild plant diversity, wider ecosystem stability with direct effects on crop production, food security and human welfare. There is clear evidence of recent declines of managed bee colonies, correlated with parallel declines in the plants that rely upon them [4,5]. In [6,7], it is estimated that out of 100 crop species which provide 90% of food worldwide, 71 are bee pollinated. In Europe, 84% of the 264 crop species are bee-pollinated and 4.000 vegetable varieties exist thanks to pollination by bees [7]. The production value of pollinator-dependent crops is five times higher than those categories that do not depend on insects [2,7,8].

Due to their importance, beehives are maintained and monitored by beekeepers that inspect in-person their hives on a prescheduled, repeated basis. This procedure is laborious and time consuming and the recurring trips to remote beehive clusters have a cost and leave a carbon dioxide imprint. Technology gradually penetrates all human activities and beehive sensing technologies are no exception and some of them have started, some years ago, to be commercially available. The sensing modalities are most commonly a weight scale for automatic weighting of the beehive to schedule optimal honey harvesting, global positioning system (GPS) tagging for theft prevention, and temperature and humidity sensors that are remotely collected and wirelessly transmitted to a remote server. These technological advances are facilitated with dropping communication fees due to competition, the availability of global SIM cards, the fact that the GPS is typically embedded to affordable communication modems and finally, the availability of free on-line services that can streamline and visualize sensors’ recordings.

The next level beyond basic information is an active research trend but not commercially established yet and relates to the health status of the beehive, mortality prevention and foraging efficacy. Questions of special interest are: is the beehive preparing to swarm? is an apiary being attacked by wasps? Is the beehive suffering from a serious pest infestation such as due to *Varroa destructor* or Nozemosis? Is the beehive queenless? How can we quantify foraging behavior? Bees’ decline is not currently attributed to a single cause, and it involves the changing climate, invasive pests and diseases spreading fast to new location assisted by the climate change, air pollution, widespread insecticide use and mismanagement of the beehives. Ongoing research in automatic beehive management is flourishing with hundreds of publications that move forward trying to answer more complex questions than optimal honey harvesting and prevention of vandalism. It would be an immense review task to mention all of them and we only mention the most recent ones related to our work. For comprehensive surveys on sensors as applied to beekeeping see [9,10] and the references therein. All these questions above and many more are not currently faced by standard, commercial solutions and different research directions involving gas sensors [11,12,13], audio or vibrations [14,15,16,17,18], new communication protocols [19,20,21,22], doppler radar in the hive entrance [23], bee counters [24,25,26] and energy harvesting techniques [27] try to offer new useful services but have to persuade the beehive manager that the extra cost is justified.

**Figure 1:**
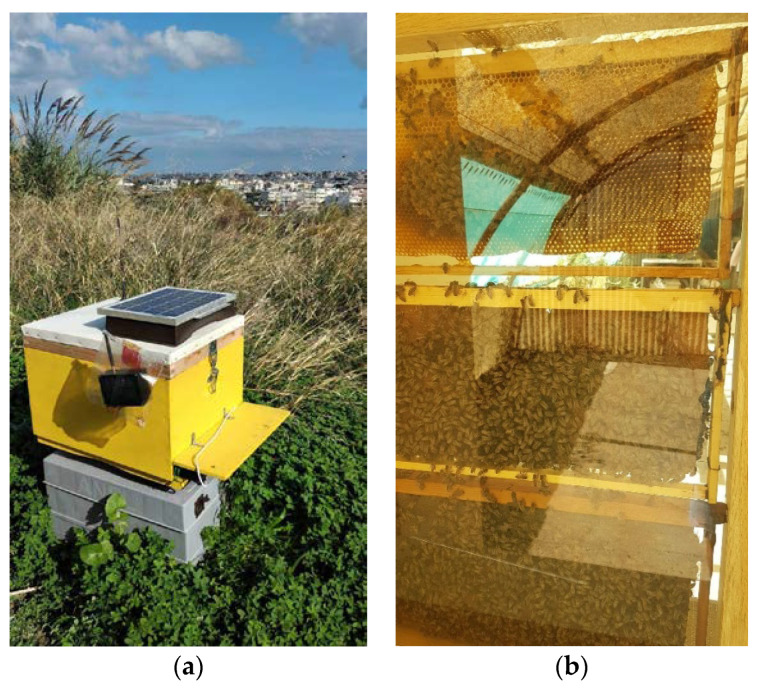
(**a**) beehives monitored in the course of this work with a number of sensors. The e-beehive in the field. (**b**) an observation beehive allows us to spot the queen and observe the patterns of activity inside it.

Our contribution focuses on health and threat status of the beehive while overcoming labor costs of manual inspections (see Figure 1) for our installations. The novelties of our research are as follows:

(A) We present an open solution that is based on custom electronics, and we do not make use of ready platforms as in many published results. Platforms such as Arduino and Raspberry Pi facilitate experimentation and are widely used in literature on beehive-related technology, but ready platforms are generic and thus not optimal in terms of power consumption and cost. We need solutions that are power sufficient because we need to avoid the manual visits to remote locations and to be affordable so that automated monitoring of health status shifts from research to commercial applications. All details are available so that it can be reproduced and its cost is assessed.
(B) We test sensor capabilities that are not the norm in commercial beehive supervision technology. Specifically, we test CO_2_ concentration and volatile compounds (TVOC) gas sensors, vibration sensors as well as a bee counter appended to typical measurements (specifically: weight, temperature, humidity, GPS coordinates, and timestamps of recording events). We design our board to sample all sensors simultaneously and create a multivariable time series that is transmitted to a cloud server.
(C) We investigate and quantify how the prediction of a single variable, e.g., CO_2_ concentration, is affected by the inclusion of additional regressors (e.g., temperature, humidity). The multivariable time series is used to run forecasting models and risk assessments [28,29,30,31], issue warnings and alert signals and make historical analysis with applied confidence intervals.

Our aim is to offer technological services that will allow beekeepers and researchers to actively participate in colony surveillance programs accurately and responsively, having less cost than the labor costs for inspecting the hive. As a result, colonies with a robustness problem or an external risk will be identified remotely earlier with greater accuracy while mitigating potential losses. The presentation of this work is organized as follows: in Section 2, Materials and Methods, we present the hardware, programming details and the prediction models. In Section 3, we present the multivariable nature of the signals that the hardware registers, their cross-correlation and an assessment of the prediction efficiency of machine learning algorithms that predict future outcomes based on past values of the multivariable time series. 

## 2. Materials and Methods

**Figure 2:**
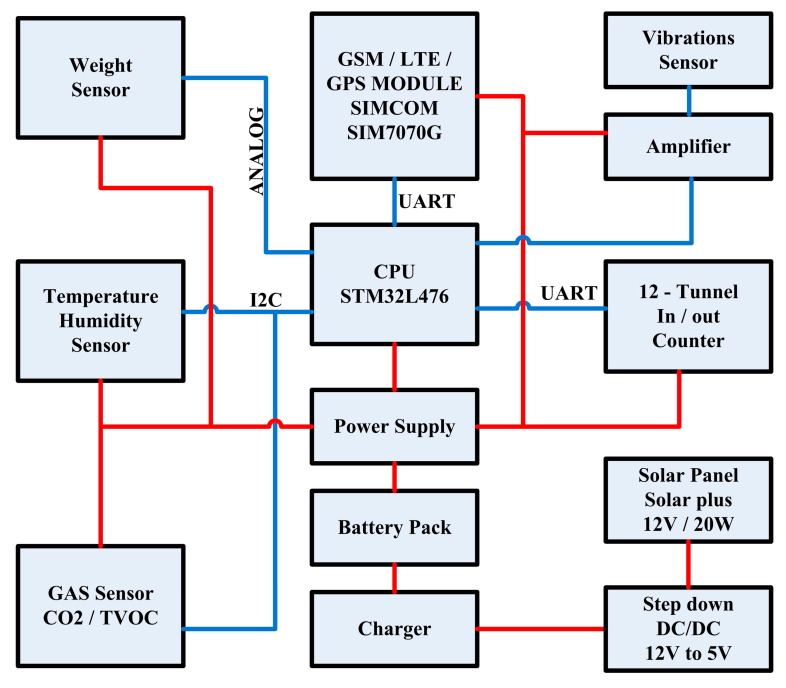
Block diagram of the e-beehive’s multichannel recorder. The system is controlled by an STM32L476RG ARM CPU of ST that simultaneously picks up the vibrations, the bee traffic in the entrance, the gas sensors (CO_2_, TVOC), the environmental variables, the vibrations and the measurements of a weight scale. All recordings, are stored in the SD card and transmitted through the LTE module. The device is powered by a 20 W solar panel that charges a battery pack of 12,000 mAh.

In this section we give a summary of the system’s design and fabrication details. A detailed list of elements for the multisensory recorder (see Figure 2) is in the Appendix A.

**Figure A1:**
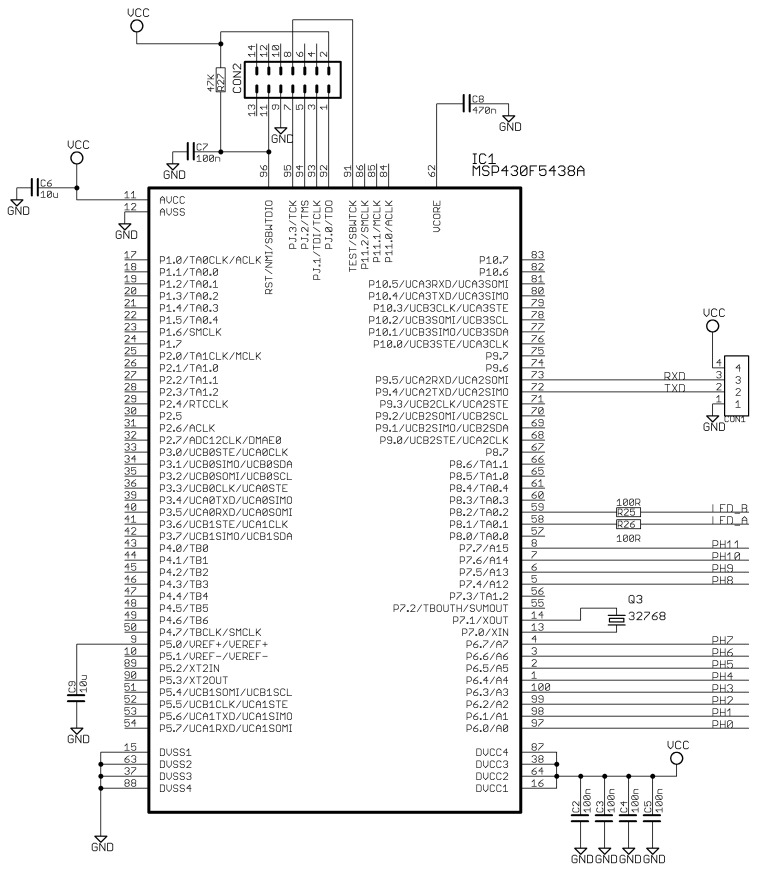
The bee counter stage processor. It is based on Texas Instruments’ MSP430F5438A microcontroller. The LED_A output activates the LEDs located on the outside of the counter and the LED_B activates the LEDs located on the inner side of the counter. PH 0–PH11 signals are the outputs of phototransistors and through them, in combination with active LEDs, the processor recognizes there is a bee in the tunnel and whether it enters or exits.

**Figure A2:**
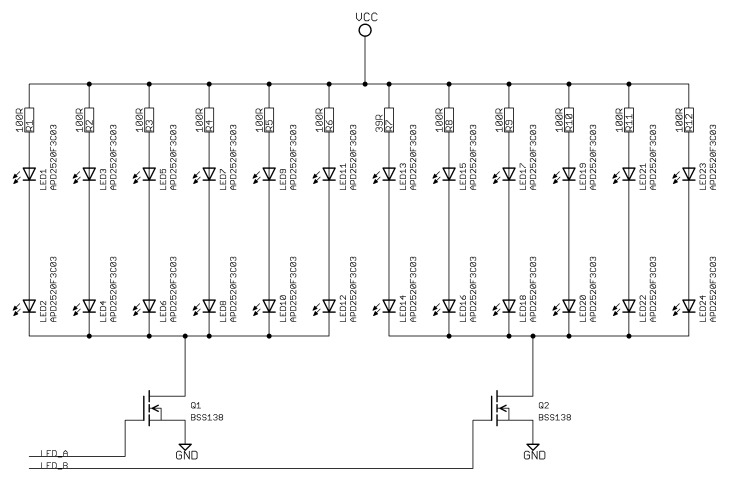
Schematic diagram of the LEDs mounted in the tunnels. When Q1 is activated, then the input LEDs emit while when Q2 is activated the output LEDs emit. By sampling at 1KHz, each set emits for 40 uSec each mSec. The total emission time for each sampling cycle is 40 uSec + 40 uSec = 80 uSec. The duty cycle is 8%.

**Figure A3:**
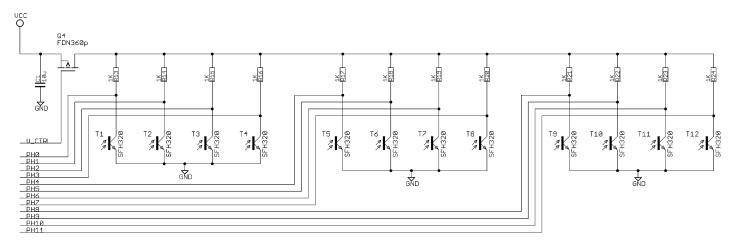
Schematic diagram of phototransistors that detect whether the infrared optical beam of the LEDs has been interrupted. In combination with active LEDs, the processor recognizes the presence or not of a bee and its direction. The Q4 controls the power supply to the circuit and is inactive for as long as it is not needed. At 1 kHz sampling, it is active 80 uSec every 1 mSec. The duty cycle is 8%.

**Figure A4:**
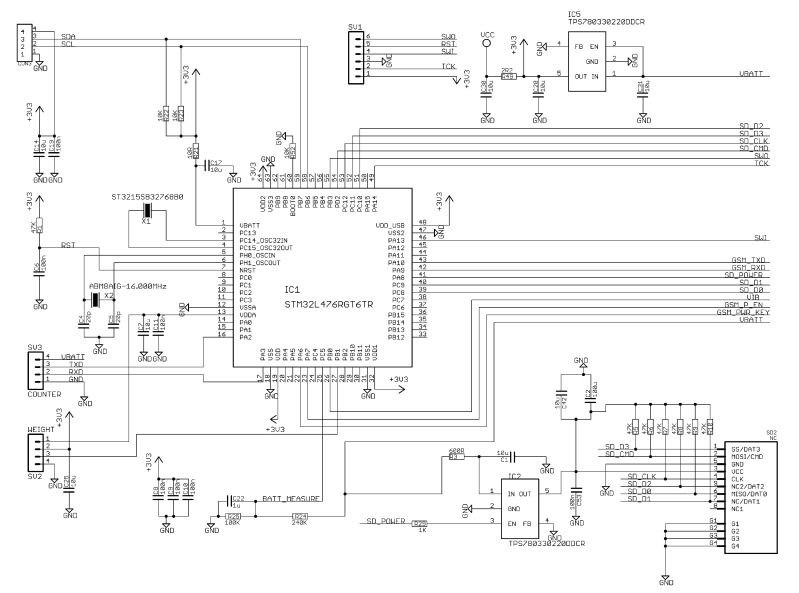
The schematic diagram of the main board of the system. It is based on the STM32L476RG microcontroller of the ST. The system is powered by a battery and the voltage stabilization at 3.3 V is done with the regulator TPS78033022 of Texas Instruments. Communication with the CO_2_/Temperature/Humidity sensor board is via the CON3 connector and the data is transferred via I2C Bus. It also has an SD card with power control to minimize consumption when not in use. Communication with the bee counter is via serial communication (UART) via connector SV3. The weight sensor amplifier is connected to connector SV2, the analog output of which drives channel 16 of the ADC. The recording of micro-vibrations is done through analog input (signal VIB, CH15 of ADC) in a file.mp3.

**Figure A5:**
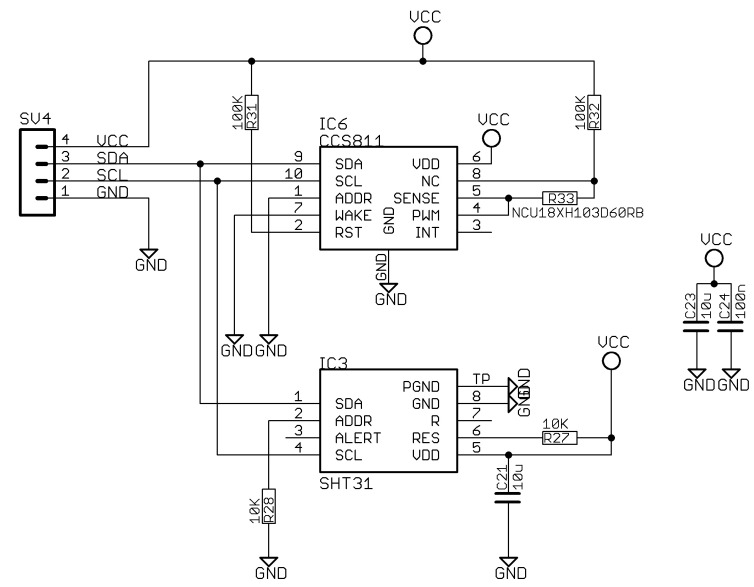
Electronic circuit of the air quality sensors (CCS811 of BioSense company) and temperature-humidity (SHT31 of Sensirion company). Communication with the main processor occurs via I2C Bus.

**Figure A6:**
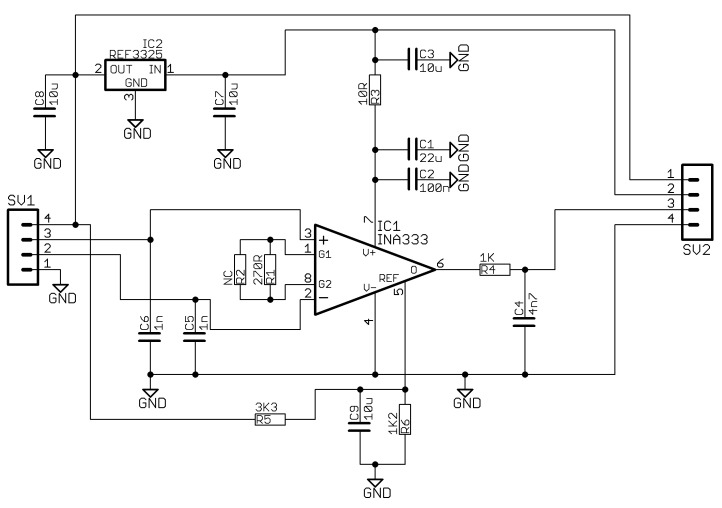
Electronic amplifier circuit of the weight sensor. It accepts input from the weight sensor and gives a voltage that is proportional to the weight. Voltage to weight conversion is performed on the system processor.

**Figure A7:**
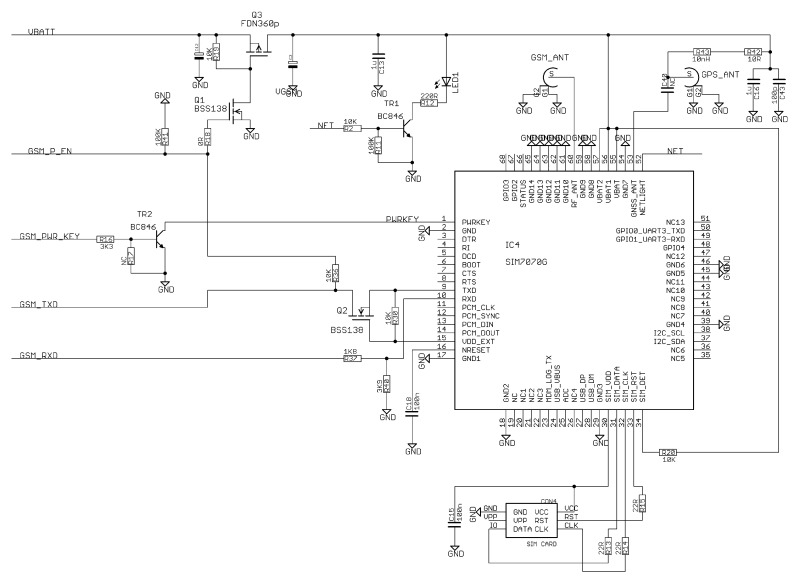
The schematic diagram of the communication circuit. It is based on simcom’s SIM7070G Cat-M/NB LTE GSM module. It connects via a UART port to the main processor and sends the data to the server with a POST request.

**Figure A8:**
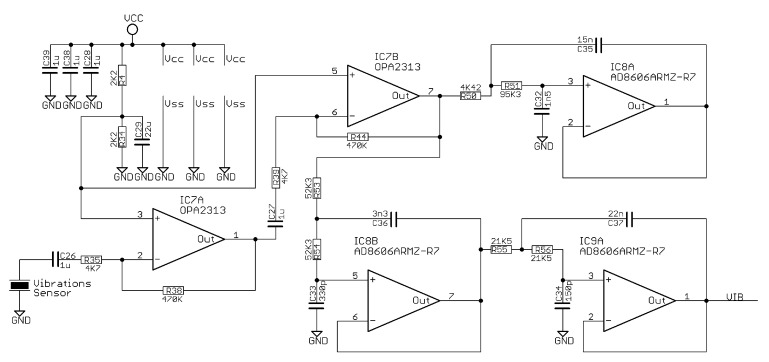
The schematic of the piezoelectric sensor amplifier. Amplifies the sensor’s output voltage microvariations (BeStar FT-35T-2.6A1), filtered with a 4KHz low pass filter whose output leads the analog input to the processor.

**Figure A9:**
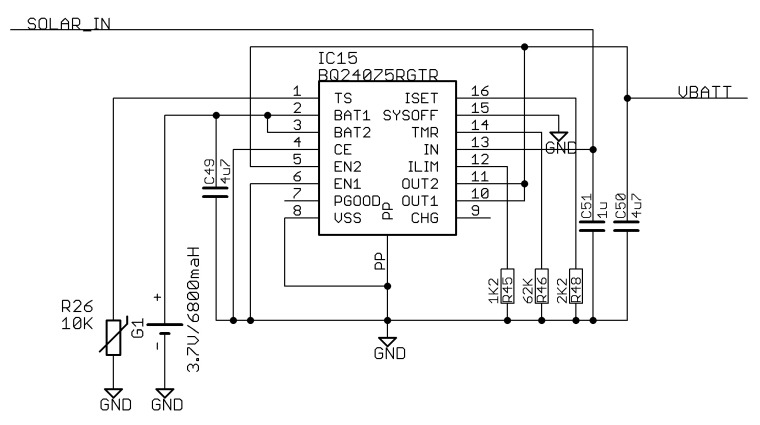
The schematic diagram of the charging circuit. It is based on Texas Instruments’ BQ24075 integrated circuit. It accepts input from the photovoltaic via DC/DC converter so that it does not exceed 5V. It has a connection input for the battery, and the output (VBATT) provides power to the system when there is a charge on the battery and/or when there is sunshine.

As seen in Figure 2, the multisensory recorder is based on a low-power microprocessor to which all subcomponents report when sampled synchronously. Namely, the sensors for weight estimation, gas concentration, temperature and humidity, the bee counter and features derived from the periodogram of the vibrations recording. The energy harvesting module is a solar panel, that charges a battery pack. All information with the corresponding timestamps, is a packet to an information stream and is uploaded to a remote server through the GSM module. The schematics are included in the Appendix A, where one can find the bee-counter (Figure A1, Figure A2 and Figure A3), the main board (Figure A4), the gas sensors (Figure A5), the weight scale (Figure A6), communications (Figure A7), vibrations (Figure A8), power regulation (Figure A9). The cost of all materials is calculated to be around 200 Euros (as per 18/12/2022).

### 2.1. The Components of the Multisensory Recorder

The microprocessor carrying out all processing and communication operations with the submodules, is the STM32L476RG (ST Microelectronics, Geneva, Switzerland). The STM32L476RG device is an ultra-low-power microcontroller based on the high-performance Arm Cortex-M4 32-bit RISC core operating at a frequency of up to 80 MHz. It reads the analog output of the weight sensor, the digital outputs of the temperature, humidity, gas and bee counter sensors. The device carries a GPS and a Cat-M/NB LTE SIM7070G GSM (SIMCOM, Shanghai, China), modem to transmit the recordings and the device’s position. 

#### 2.1.1. The Gas Sensor

We have embedded the integrated circuit CCS811 Ultra-Low Power Digital Gas Sensor ScioSence that delivers CO_2_ from 400 ppm to 32,768 ppm and TVOC concentrations from 0 to 29,206 ppb through the I2C Βus. 

##### CO_2_ Concentration

As regards the concentration of CO_2_ in the atmosphere, this year, the barrier of 410 parts per million (ppm), a unit of measurement used to assess air pollution, has been exceeded. Before the industrial revolution, the level of CO_2_ was permanently around 280 ppm. CO_2_, also a byproduct of breathing, is toxic at high concentrations, so regulating CO_2_ inside the beehive is an important function of the colony [32]. Aspects of controlling the concentration of CO_2_ in the hive may reveal information about the health of the colony. In this study, we measured CO_2_ concentrations of the hive at intervals of 5 min, while the ventilation characteristics of the hive changed every few days and we visualized the data. A concentration below 417 ppm would be an indication of a sensor’s malfunction and would create an alert message. A large concentration of >17,000 ppm would also lead to an alert as it would indicate a problem with ventilation and this is where we set a boundary.

##### TVOC Concentration

Total volatile organic compounds (TVOCs) are responsible for the odor of perfumes as well as pollutants. TVOCs play an important role in communication between animals and plants, e.g. odors to attract pollinators, avoid predation and even interactions between plants. Some TVOCs are dangerous to human health or cause harm to the environment. A bee colony is an effective environmental sampling device for volatile organic compounds (VOCs) in a complex ecosystem. They are also associated with a wide range of compounds that are environmental pollutants. Flower fragrances are usually mixtures of volatile substances of various chemical groups. On the other hand, fossil fuel components, industrial solvents, pesticides also fall into this category. TVOC values in beehives can serve as biomarkers. Our sensor records measurements in ppb (parts per billion). It does not have the ability to single out the source of VOCs. It has been supported that there is a link between VOCs and *Varoa destructor* [33]. However, TVOC measurements cannot realistically be expected to give a direct indication of the present *Varroa* population, and the observed correlation or dependence can be attested as an indirect indication of changes in the behavior of the beehive due to the imposed stress of the growing mite population in the bee colony. We include TVOC measurements in our system more as an indication of stress and deviation from normality and we use it as an extra covariate with the rest of the measurements.

#### 2.1.2. Vibrations

We chose to record vibrations, over audio through a microphone as bees communicate primarily through vibrational and chemical signals and less through audio. Vibrations pass through the wooden substrate; piezoelectric transducers are more durable than microphones in the presence of humidity and propolis deposition does not stop vibrations from being propagated. A metal waveguide is attached to a piezoelectric transducer (BeStar FT-35T-2.6A1), that is based on a piezo-ceramic disk. We chose this sensor mainly for its frequency response (i.e., 1–10 kHz) and high sensitivity. The output of accelerometer is amplified and filtered. The analog filter output is converted to digital words in 12-bit resolution at 8 kHz sampling rate using the internal ADC of the microcontroller. The sampling frequency, record duration and other initialization parameters are read once from the SD card during powering-on and are configurable through the server.

Bees communicate with vibrations as well and vibrational activity is an indicator of an active queen. A change in audio and vibrations has often been reported as a sign prior to swarming [15]. Therefore, the vibrations sensor is a useful add-on for the e-beehive, as a modality that contributes to the assessment of beehive’s health. The collective vibrations can naturally be converted to an audio signal. The spectrogram is a time vs. frequency representation of sound showing the spectral content of the audio as it evolves in time. Once we have the frequency content, one can search in the frequencies for specific communication signals such as the ‘whooping signal’ [18], ‘tooting’, ‘quacking’, ‘piping’, ‘waggle’, ‘tremble’ [34,35,36,37,38,39,40]. Note that the sensor picks up the collective vibrational pattern of, possibly, thousands of bees and, therefore, it is not a straightforward task to pinpoint a specific behavioral audio-signal. In [41], the author states that a rise in acoustic signal magnitude at 255 +/− 35 Hz was indicative of swarming or that the queen was failing, whereas a hiss at 3000+ Hz in response to banging on the hive could determine that the colony was healthy. In Table 1, we collect from the literature the most important vibrational patterns and their alleged role. There is some controversy in the literature over the role of the vibrational pulses of main interest to our present study and, sometimes, over the frequency range.

#### 2.1.3. Temperature and Humidity

The humidity and temperature sensor consists of Sensirion’s SHT31 integrated circuit. It has a measurement accuracy of 0.2 °C at temperature and ±2% RH at humidity. It measures a temperature of −40 to 125 °C and a relative humidity of 0 to 100%. Communication with the microcontroller is carried out by the I2C Bus.

##### Temperature

The effect of temperature variations on bees well-being is significant: hives without bees record a lower average temperature and a wider temperature range than hives containing live bees. Hives without bees reach the maximum temperature earlier than hives with bees, regardless of the strength of the colony. Data from sensors come from the brood that was found to be more closely connected to the health of the colony and this is where we place the sensor. Temperature is a critical factor for colony health and is actively regulated by *A. mellifera* using heating and cooling behaviors to maintain a constant temperature within the range of 34–36 °C. The maintenance of "hive homeostasis" is a good indicator of the health of the colony, its condition and even its activity. In many experimental published papers, temperature detection showed correlations between the health of the colony and its strength. The boundaries for alert in our system were set to 8 °C as a lower bound and to 37 °C as the high one, beyond which there is significant danger for the beehive.

##### Humidity

Bees have developed adaptive mechanisms to cope with environmental fluctuations, including those of humidity in the brood. In addition to the humidity of the environment, bees create moisture through processes such as the introduction of water, liquid nectar and even through their breathing. While it is important to maintain a certain level of humidity, it is important that moist air does not condense on the inner walls of the hive or on the frames. The balance in the hive is maintained when the humidity fluctuates between 53–70% with variations in external humidity of 42–100% [47]. In temperate climates during the summer, the humidity in the hive does not seem to be a problem due to the fact that the outside air is warmer and drier, so passive air circulation is more efficient and is helped by larger inlets. On the opposite side, during the winter and spring months in temperate climates, the humidity of the environment tends to be high due to rainfall and lower air temperatures. Interestingly, the majority of the moisture that is in the hive during the winter is the direct result of bee metabolism. Relative humidity at high levels implies that there is a risk of condensation or that the beehive is not strong enough to regulate humidity (health hazard), and this creates a useful alert for beekeepers to increase the ventilation of the hives. The boundary for alert is set to 90% RH.

#### 2.1.4. The Weight Sensor

The weight sensor is made with the single point load cell CLZ601 of Standard Loadcells. Its output is enhanced by the instrumentation amplifier INA333 and drives the analog input to the microcontroller. A healthy hive with floors can contain up to 60,000 worker bees and can weigh over 50 kg when loaded with honey. Thus, an effective way to monitor the health of the bee colony and honey production is to weigh the hive. Weighing the hive gives us a very good idea of how many kilograms of honey are inside. When the measurement during the harvest period reaches a plateau, it is an indication that at that time, a harvest must be made. Keeping an eye watch for the optimal harvest moment is a main reason for the existence of the weight scales that are usually the main commercial component. Another important reason is the one linked to the health of the hive: it is important to know how much honey the bees have during the winter. Bees cannot go out in the cold and depend on their honey to stay fed and warm during winter. Depending on where the hive is located and how heavy the winters are, bees need a certain amount of honey reserves to survive the winter. Weighing the hive in the fall will tell us how the honey stocks of bees are and whether they should start feeding them syrup so that they live until spring. Moreover, the regular transmission of weight measurements also helps in the detection of swarming, when a sudden and significant weight loss is observed. The lower bound of an alert is signal is set on the weight of the beehive without bees.

#### 2.1.5. The Bee Counter

The bee counter is based on a simple construction of 24 light emitting diodes (LEDs) and 12 phototransistors. Each phototransistor is illuminated from two LEDs (one at a time) in a row at the entrance and one at the exit of each tunnel. So, with the succession of shading in phototransistors, the processor discerns the direction of the bee. The Texas instruments MSP430F5438A has been used for the CPU. It is low power and has the appropriate analog inputs for connecting the phototransistors. It communicates with the system’s main processor (STM32L476RG) via a UART port to send the measurements and receive the settings it will need. 

The ability to accurately measure the number of bees in a hive is not only important for scientists, but also for beekeepers. Beekeepers are provided, for example, with the means to assess foraging activity and observe the behavior of swarms and the development of the colony. If abnormalities occur (e.g., sudden drop of bee counts) bee counters helps to find the underlying causes (e.g., widespread use of insecticides). More probable causes need to be ruled out first, such as very bad weather. EFSA (European Food Safety Authority (EFSA), [48] p. 156–163) states the overall natural bee mortality rate at 3–4% so a significant difference in entry–exit values at the end of the day is a reason to create an alert warning. As workers usually fly only during the day and return to the hive in the evening, the 24-h observation intervals are appropriate and are recommended by EFSA as a reference (European Food Safety Authority, [49]). Note that bees can follow a pattern of in and out several times per day and, therefore, the counts do not correspond to the exact number of bees and what is only important is the difference between the in and out counts at the end of the day. In our work, the bee counter is automatically nulled every midnight and counts the accumulated number of passages in and out on a 24 h basis. That is, at 00.00 every day, the meter is reset and cumulatively adds up until the next day at midnight (cumulative counting). The daily loss, i.e., the difference between incoming and outgoing bees, can be used to assess colony health, environmental impacts and to draw conclusions about the effect of agricultural pesticides on bee colonies. 

#### 2.1.6. Power Supply

The power supply of the system comes from a rechargeable lithium battery 3.7 V/12,000 mAh (INR1865030 Q, pack 4 × 3000 mAh) which is charged by a 12V, 20 W photovoltaic panel using the BQ24075 (Texas instruments) charging chip. 

### 2.2. Programming

**Figure 3:**
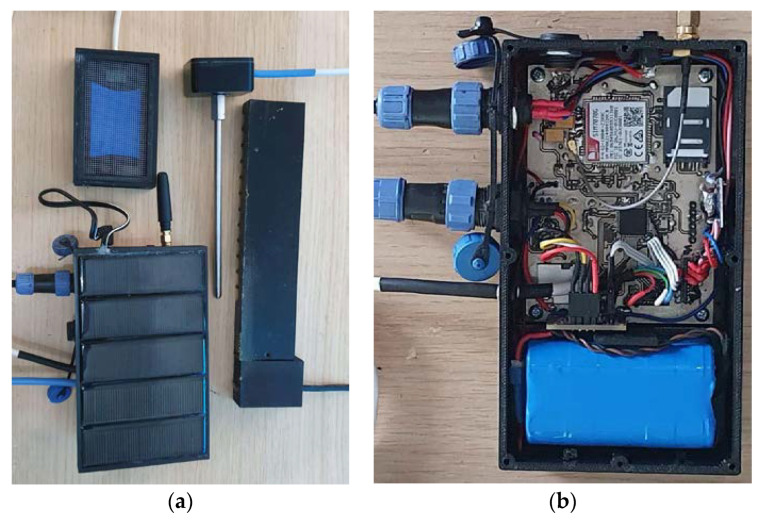
(**a**): the central monitoring unit in the center of the picture with some of the sensing modalities attached. All sensors are sampled simultaneously, and their readings are collected from the main CPU. (**b**): a closer look at the electronics board prototype. One can discern the CPU in the center, the GPU and communications modem on top, the SD card on top right, the battery on the bottom that connects to the solar panel.

The board (see Figure 3) is programmed in C/C++. The software is written in C language using the IAR Embedded workbench. The programming of the flash memory was carried out using the ST-Link V2 programmer. The code initialization was done using the STM32CubeMX of ST. For programming the peripheral sub-components such as the SD and ADC we made use of the STM32 HAL Drivers. The server manages the data collection and timestamping, GPS registration and storage process of submitted audio recordings. Moreover, the administrator can manage and customize the data fields and collection process as well as communicating commands to the sensor nodes. The Cat-M/NB LTE GSM modem of the beehive once connected to the mobile provider, can have internet connectivity. 

The device sends measurements to the webserver using a POST request. At this point, the trap inserts its data as parameters for the page that it wants to access. Once the HTTP request reaches the web server, the latter receives the data from the request of the trap (via the appropriate code, written in PHP) and logs the information in the database. The measurements on the server are sent with a JSON format and a timestamp. The communication of the module with the main microcontroller of the system is through UART with AT commands. The code has been optimized for minimal power consumption and several parts of the hardware are activated only when they are needed to function and hibernate straight after performing their task.

### 2.3. Prediction Models

A time series is a sequence of data taken over time and chronologically ordered. In our case, the spacing between the datapoints is uniform (every five minutes) and the time series is multivariate due to the presence of many sensors sampled at the same time. The time series has encoded temporal information, between present and past data and due to the possible dependency between different sensing modalities. In the context of beehive monitoring, we are interested in predicting when the beehive is evolving to an abnormal state, to anticipate and prepare for, respond to and recover from these events. There are dozens of machine learning techniques that can be applied to a time series problem, to interpret the series and extract information about underlying relationships between the target series and the regressors (e.g., bee counts or CO_2_ concentration), and this work is not about finding the optimal one. Deep, learning techniques, transformers, recurrent neural networks (RNNs), multivariate linear regression (MLR) and a long short-term memory neural networks (LSTM), gradient boosted trees, ARIMA, support vector machines, autoregressive models are typically used in time series’ forecasting [28,29,30,31]. In this work we are mainly interested in high interpretability, and therefore we chose an additive regression model, with three principal components (trend, seasonality and cyclicity). A trend is a long-term increase or decrease in the data, seasonality relates to fixed cyclical patterns (e.g., activity during different hours) and cyclicity relates to long term fluctuations that in our case, have to do with the climate, use of insecticides etc. For means of comparison, we also present results from XGBoost which is an optimized distributed gradient boosting library that also provides interpretable results. We want the forecast procedure to be a part of a decision support system (DSS) where predictions of sensor values provide the beekeeper with the opportunity to respond timely [44]. Without forecasting, the e-beehive alerts only when a sensor’s measurement violates the bounds set by apiculture’s literature. These bounds are very loose, reflecting extreme conditions, so that false alarms do not emerge often. When one has historical data, one can make predictions of lower and upper bounds of the predictions’ uncertainty interval and compare whether the actual sensors’ measurements fall within this interval. Outliers are defined as any sensor’s value outside of these predicted intervals. Therefore, tighter bounds on what is considered normal beehive behavior can be derived. Tighter bounds provide the means for an earlier alert on abnormal behavior.

## 3. Results

### 3.1. The Signals

In this section, we describe the nature of the signals we record and what tools can be used to process them to primarily extract alert signals for the beehive manager. Here is the full list:

(1) Time stamp of events. Measurements taken every 5 min on a 24/7 basis, for all sensors simultaneously.
(2) GPS coordinates that are used to localize the beehive on a map, on the server’s part and as a theft prevention measure.
(3) CO_2_ concentration inside the hive (parts per million—ppm).
(4) Total volatile organic compounds concentration TVOC inside the hive (parts per billion—ppb).
(5) Temperature inside the beehive (°C).
(6) Relative humidity in the interior of the beehive (% RH).
(7) Weight (Kgr) of the beehive (including honey, bees and pollen).
(8) Incoming bee counts.
(9) Outgoing bee counts.
(10) Five sec vibrations recording from which features are extracted (e.g., energy in specific frequencies that are deemed important e.g., piping, tooting, tremble, whooping signals).
(11) Quality of signal communication (used for telemetry at the server part).
(12) Battery charge level (used for telemetry at the server part).

**Figure 4:**
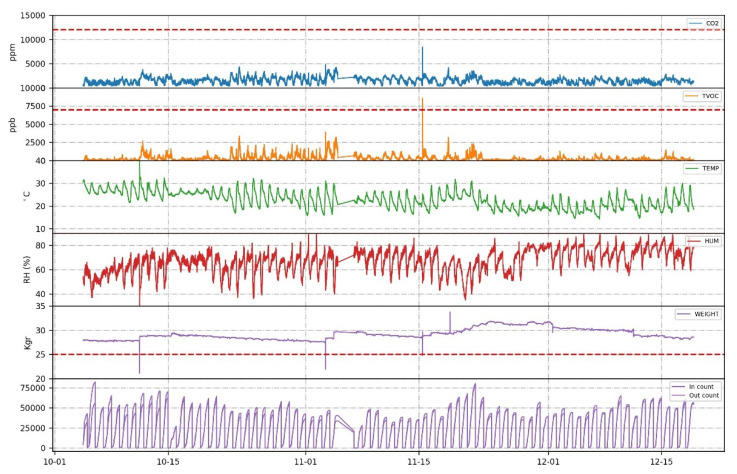
A part of the multivariable time series. Forming sensory data this way allows better forecasts due to complementarity of informational queues.

The sensory data are streamlined in Figure 4. The dashed, superimposed horizontal lines denote the signal boundaries that are derived from the apiculture literature. If any signal surpasses these boundaries, a corresponding alert message is issued from the server to the user. Another observation is that humidity and temperature fluctuations are not large, and this implies that bees self-regulate these parameters inside the hive. Weight change is slow, and one can see the days of feeding treatment where the weight spikes and the slow reduction of the weight during winter. Finally, we see how the numbers of incoming and outgoing bees are almost the same. During the first week of operation, there have been some shading problems with the bee-counter that have been corrected by proper action (see Figure 4-bottom until 10/15/22 in the x-axis). The contour analysis of the CO_2_ concentration data found evidence of significant CO_2_ cycle periods both within and outside a 24 h period. Bee colonies maintained strong daily CO_2_ concentration cycles, with average maximum concentrations >4000 ppm, even in conditions of increased ventilation, indicating that the management of the CO_2_ concentration is important to be monitored. Daily changes are not correlated with changes in weight (because this remains approximately constant in the week) and temperature of the beehive and the maximum and minimum are different every day.

**Figure 5:**
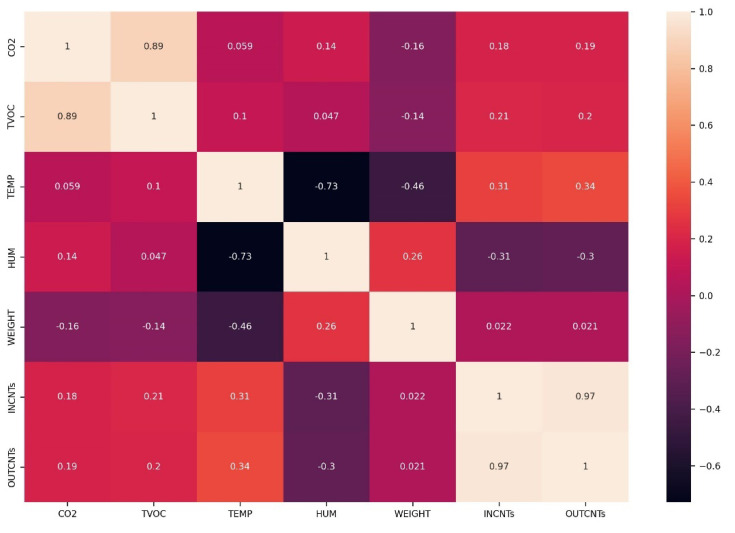
Cross correlation heatmap of all sensory inputs. TVOC and CO_2_ show high correlation and humidity and temperature strong anti-correlation. In and out counts are highly correlated.

In Figure 5 we derive the cross-correlation matrix between all sensory signals. Cross-correlation is used to compare the variables of multivariable time series and objectively determine how well they match up with each other and how important one is in the co-interpretation of the targeted data. We immediately derive from the heatmap of the correlation matrix that TVOC and CO_2_ are highly correlated whereas humidity and temperature are anti-correlated which is expected, as when the temperature rises, humidity is reduced. Incoming and outgoing bee counts are highly correlated as they should be. 

**Figure 6:**
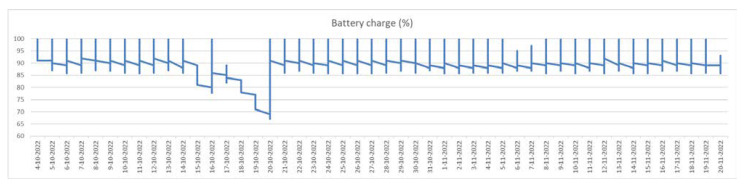
Daily balance of power consumption using a 20 W solar panel. Note, the sufficiency of the power-supply scheme, even in winter time, for hourly emissions of data.

We claim that our configuration is power sufficient and a 20 W solar panel charging a rechargeable pack of 4 × 3000 mAh batteries is sufficient for unobtrusive operation even in winter time. We cannot derive an explicit closed-form solution for power sufficiency because it depends on weather conditions that affect the solar energy harvesting of the panel. However, the device has a configurable, through the server, data transmission, hourly pattern, that can change if needed to a sparser report plan to save power if there is no sunlight for a long period. In Figure 6, we have a chart of the power balance for a constant operation of almost two months during winter. During night, the e-hive has the minimum charge that most of the time does not fall under 85% of full capacity and peaks sharply to 100% during the day while falling linearly during the day in every emission. Since, for a period of two months during winter, the energy sufficiency did not drop below 65% while emitting 24 times per day, and the monitoring would be still adequate with one transmission per day, we suggest that the beehive is power sufficient.

### 3.2. The CO_2_ Concentration

**Figure 7:**
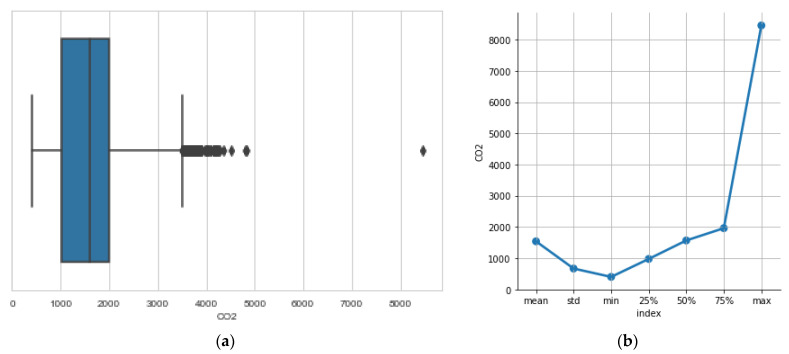
(**a**): boxplots pinpoint outliers in the recordings of CO_2_ concentration. They also show most probable value of concentration and the spread of values. (**b**): descriptive statistics: the mean value, the spread denoted by std (standard deviation), the min and max values are of special importance to the interpretation of the data.

In Figure 7a we see the boxplot of the CO_2_ sensor. A boxplot is a tool in descriptive statistics that captures the summary of the data efficiently, for graphically demonstrating the median, spread and skewness groups of numerical data. The boxplot summarizes sample data using 25th, 50th and 75th percentiles. We use it to easily locate outliers, i.e., values of the sensor that are significantly higher or lower than the median value. In Figure 7, we immediately spot a large value over 8000 ppm which is, however, not repeated. Concentrations around 4000 ppm are also evident but, again, are not persistent.

### 3.3. The TVOC Concentration

**Figure 8:**
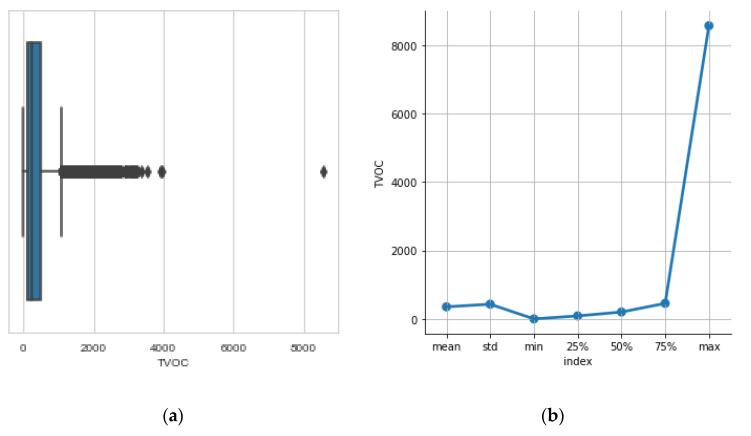
(**a**) boxplots pinpoint the outlier value of >8000 in the recordings of the TVOC. (**b**) TVOCs have a smaller spread and lower values compared to CO_2_.

**Figure 9:**
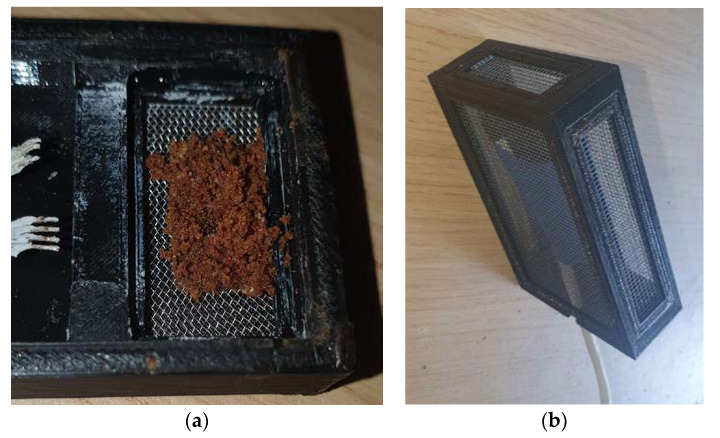
(**a**) the bees covering the gas sensor with propolis on the left. We left only partial coverage as it was totally covered. (**b**) new, gas-penetrated box houses the gas sensor and placed in the beehive in such a way that not all sides can be covered by propolis.

In Figure 8, we use the boxplot as a tool for spotting outliers in the data. In Figure 8b we see descriptive statistics: the mean value, the spread denoted by std (standard deviation), the min and max values are of special importance to the interpretation of the data as for TVOC they show us small spread and pinpoint the outliers. Note that during operation in the field, the server received very low values of CO_2_ and TVOC concentrations which indicated a malfunction. The visit in the beehive revealed that bees had deposit a large quantity of propolis on the sieve of the gas chamber and blocked the air circulation around the sensor (see Figure 9a). We knew that was a possibility, but it was also a chance to detect malfunctions. The problem was solved by placing the sensors inside a more versatile box covered with a sieve that was placed in the beehive in a way that it was not easy to block all its sides (see Figure 9b). 

### 3.4. The Weight

On Figure 4, fifth row, we notice that the weight within October–November has not changed significantly. Alert signals are placed on the difference of the values to catch an abrupt change and only on the weight slightly over the weight of an empty hive.

### 3.5. The Temperature

**Figure 10:**
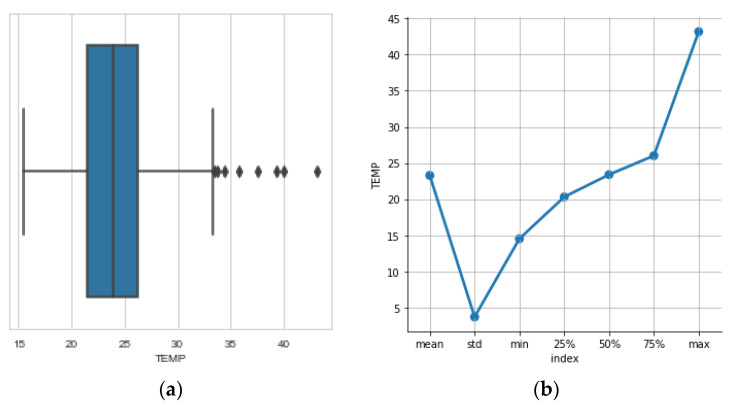
(**a**) boxplots pinpoint outliers in the recordings of temperature (°C) that are deemed dangerous for the health of the beehive if they are prolonged. This is not the case here. (**b**) mean, std, min and max values are valuable to look out for normal values for temperature fluctuations and outliers.

The behavior of the temperature variable is immediately evident in the boxplot of Figure 10a and the descriptive statistics of Figure 10b. The mean temperature is around 23 °C with a small spread around ±3 °C. The lowest ambient temperature range recorded on any given day was 15 °C where the bees come close together to increase the temperature of the hive and the highest 43°C (a single outlier) where the bees ventilate vigorously to decrease temperature. Daily temperature ranges inside the beehive but the standard deviation is small, as depicted in Figure 10. The boxplot in Figure 10 and its associated descriptive statistics are used to derive the spread (see std) and the outliers in temperature.

### 3.6. The Humidity

**Figure 11:**
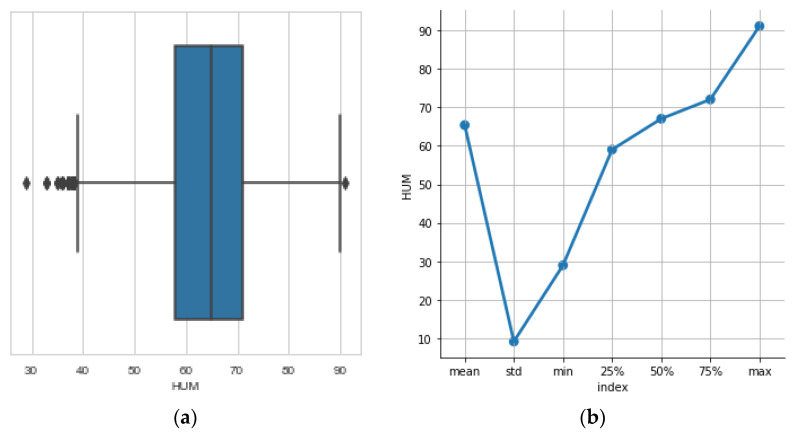
(**a**) boxplots pinpoint outliers in the recordings of humidity. Beehives must not have measurement near 90% RH for a long time as this implies condensation. (**b**) the mean and std values show that the humidity levels are normal inside the hive. Special attention should be given to the 90% RH that is, however, a non-persisting outlier.

A boxplot in Figure 11 is used to quickly grasp the essence of this variable. Humidity levels are quite uniform and representative of what we usually observe in healthy queen colonies: 40–70% RH. A strong colony has a much smaller overall variation in humidity and its pattern is not related to the ambient temperature, thus indicating a significant degree of self-regulation. We place an alert limit when the humidity exceeds 90% which means that we have water condensation inside the hive. In the descriptive statistics of Figure 11b we derive the spread around the mean and the outliers in humidity (>90% RH).

### 3.7. The Vibrations Sensor

**Figure 12:**
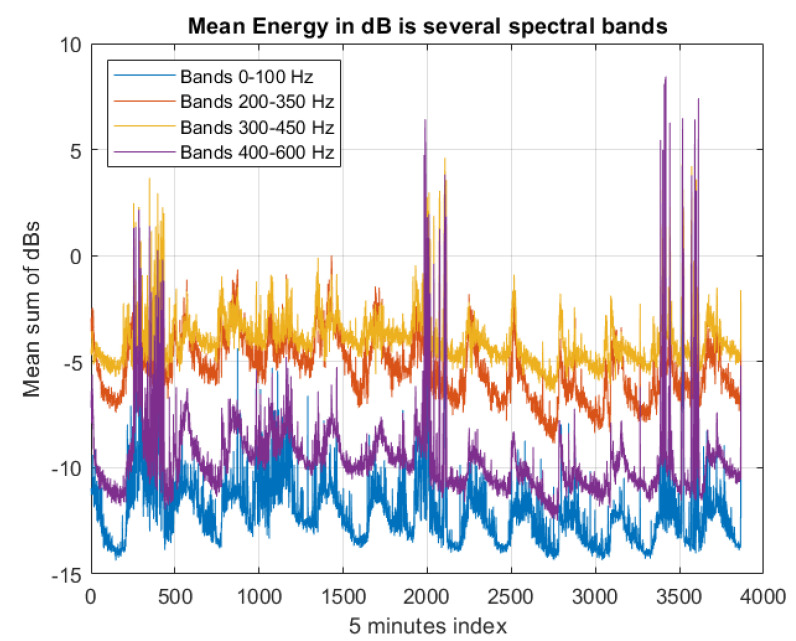
Descriptors of energy bands. The spectrogram is a time-frequency 2D representation in dB. The descriptors are summing the energy in the corresponding bands 0–100Hz, 200–350 Hz, 300–450 Hz, 400–600 Hz.

**Figure 13:**
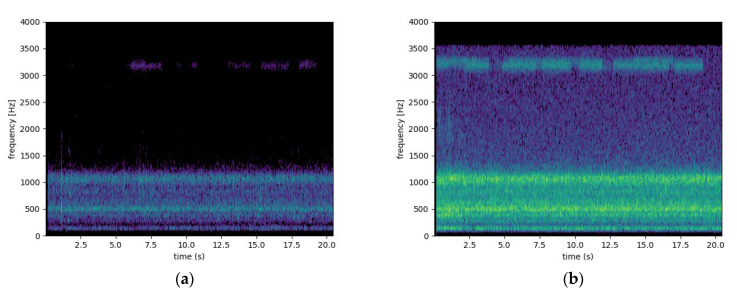
Vibrational signals taken from a piezoelectric transducer from within a hive with several thousand bees. (**a**) a morning recording. (**b**) an evening recording.

The frequency content of a vibrations recording is typically analyzed by using the short time discrete Fourier transform (DFT). Since we are mainly interested in specific frequency bands associated with the aforementioned sounds as summarized in Section 2, Materials and Methods, the Goertzel algorithm can also be used. The Goertzel algorithm is a technique in digital signal processing for efficient evaluation of the individual terms of the DFT. However, in this work we extracted directly from the spectrogram (short-time DFT), the energy of the specific spectral bands that in literature they are reported to be associated with bee sounds, and we append these band-energies as features to the main time series in Figure 4. We subsequently extracted 4 bands as feature descriptors of vibrational activity: 0–100 Hz, 200–350 Hz, 300–450 Hz, 400–600 Hz (see Figure 12). See also Figure 13 for the spectrogram of two characteristic recordings.

We have also recorded the vibrations from inside a beehive every 5 min for two months and appended the corresponding spectrogram into a video file (see Supplementary Materials).

### 3.8. Error Measurements of Prediction Models

#### 3.8.1. Prophet: Additive Regression Model

The additive regression model is stated in Equation (1).

(1) $$y\left(t\right)=g\left(t\right)\times \left(1+s\left(t\right)\right)+r\left(t\right)+\varepsilon$$

where *y(t)* is the prediction, *g(t)* the trend, *s(t)* the seasonality, *r(t)* the regressor and *ε* a white noise term.

The multivariate time series allows us to peak any variable of interest as the targeted variable to predict. Moreover, Prophet also gives the option to add extra regressors to the model which, in our case, are the rest of the variables excluding the targeted variable.

**Figure 14:**
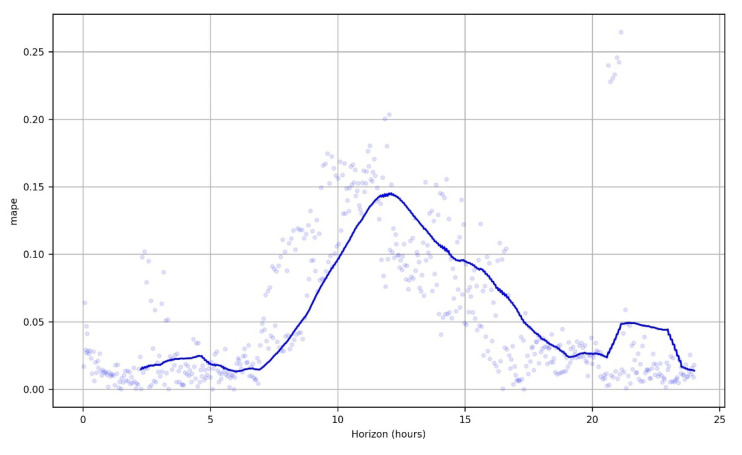
MAPE over a horizon of 1 day (5 min × 12 × 24 data points to be predicted) in the humidity variable.

The train set is the whole dataset excluding the last two weeks that are retained for the test set. We use the mean absolute percentage error (MAPE) to measure the final error of the model over the test period for the variable humidity (see Figure 14). We used as additional regressors all the other variables except humidity. MAPE metric gives a measurement of the relative error, as a percentage with respect to the real data, so its interpretation, which is what interests us now, is more intuitive than that of the mean square distance between the prediction and the actual data (MSE). We accept a MAPE below 10% as good. We also measure how well our model fits the variability of the data with the metric R^2^. Table 2 summarizes the results. Note that if we do not use additional regressors and base our prediction on just the humidity variable then the MAPE in training and test is around 6 and R^2^ in the test set is 19.66.

**Figure 15:**
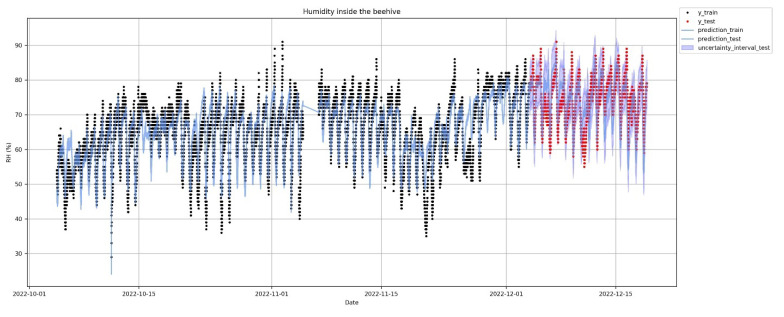
Prediction of humidity inside the hive on the whole dataset. Prediction starts after ’04–December–2022’. In red, the actual values and in blue the prediction and the uncertainty intervals.

**Figure 16:**
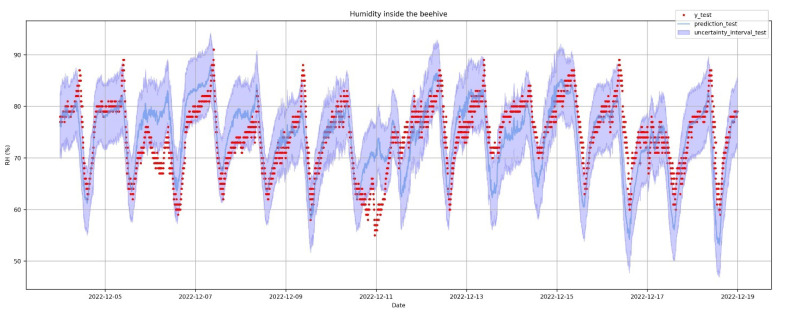
Prophet: prediction of humidity inside the hive. Zooming in the test period.

MAPE of the test set is low, and R^2^ in the test is also very high, which implies that the model explains the variability of the data well but cannot capture abrupt spikes of the data which are typical in the volatility of humidity. This is obvious in Figure 14, where most of the time the MAPE is at 5%, but in spikes it becomes significantly larger. Comparing the results in the test set with those in the train set, shows that there is a moderate overfitting, since the results in train stage are better than in test phase but not far away. Figure 15 is very revealing because it provides a lot of information at a glance regarding the accuracy of the forecast. Figure 16 presents in more detail the predicted humidity levels along with the lower and upper bounds of the uncertainty interval. These bounds of uncertainty can be used to spot an outlier when the actual measurement is outside these predicted bounds. One can compare these bounds with the flat alert thresholds as set in Figure 4 on the humidity time series and see that these of Figure 16 are much tighter.

**Figure 17:**
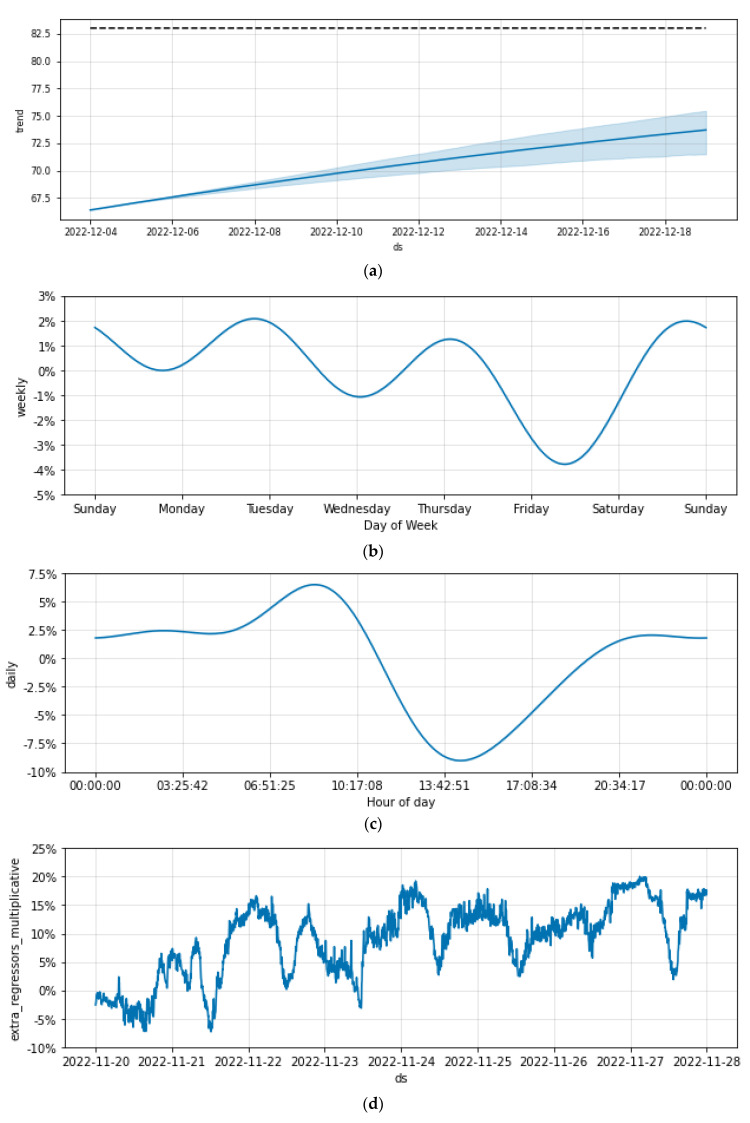
Decomposing the time series in trend (**a**) and cyclicity factors (**b**,**c**). Practically, no weekly trend is detected in (**b**), and a daily strong cyclicity of humidity detected between day and night hours in (**c**). In (**d**) the role of extra regressors is quantified.

Prophet allows decomposition of the series so that one can see the trend and the cyclicity of factors (see Figure 17). The trend in Figure 17a shows that the humidity is increasing which is normal for the winter. The weekly seasonality as depicted in Figure 17b is very low as expected (as the bees do not discern days) whereas the daily cyclicity in Figure 17c shows that humidity is increasing in the evening and is lowering in the morning. The latter is also reasonable as it relates to environmental humidity and the exit of the worker bees in the morning and their return in the afternoon.

#### 3.8.2. The Xgboost Regression Results

**Figure 18:**
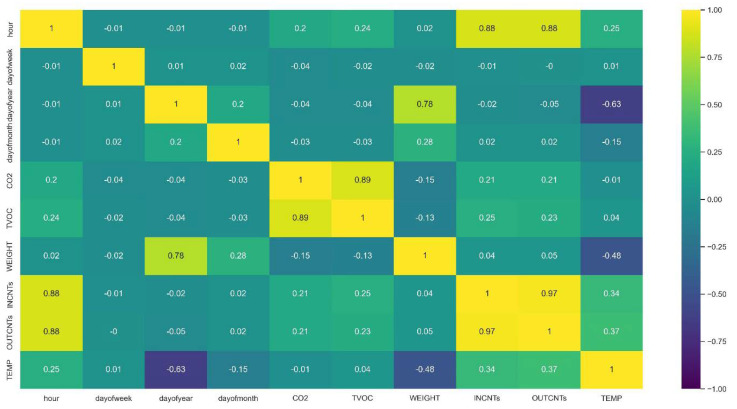
Correlation of features. The hour feature affects CO_2_, TVOC, TEMP and strongly the bee traffic in the entrance. The day_of_the_year variable affects weight. CO_2_ and TVOC are highly correlated and in and out bee counts as well.

XGboost is based on features and feature engineering [50]. In our case, the features are the sensor’s readings appended to several variables that have to do with the hour and date. In Figure 18, we see the heatmap of the correlation matrix ranging from 0–1 (i.e., 0–100%). The same correlations as in Figure 5 are derived but augmented with some other interesting correlations related to time (i.e., the hour variable, day, day of the week, day of the month) and how time is correlated with variables such as traffic in the entrance, TVOC and CO_2_ levels. The day of the year is also well correlated with weight.

**Figure 19:**
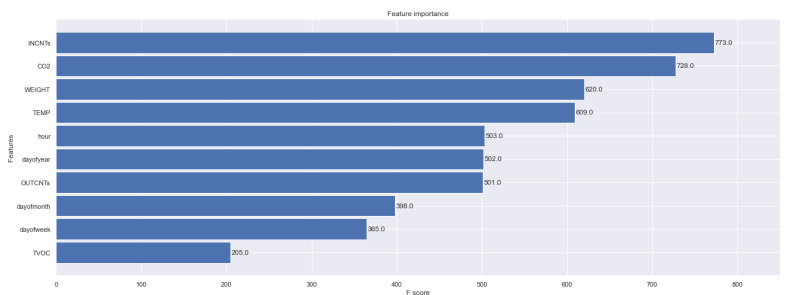
Feature importance on the regression task of forecasting the humidity level inside the beehive using gradient boosting trees (XGBoost).

**Figure 20:**
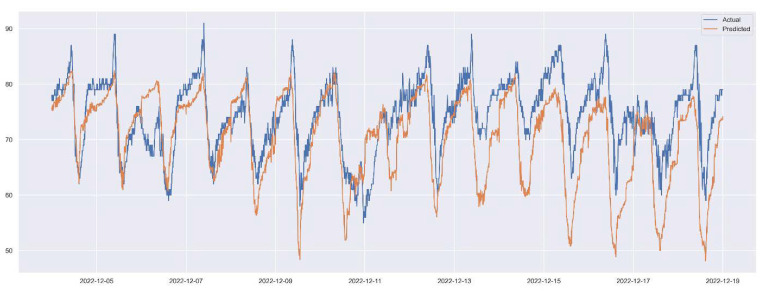
Prediction of humidity inside the hive using gradient boosted decision trees. Zooming in the test period.

XGBoost allows us to validate feature importance and, interestingly, it reports that CO_2_, traffic in the entrance and temperature have high effect on the prediction of the humidity variable (see Figure 19). Finally, we derive the same error metrics in Table 3 and the forecast of humidity on XGBoost in Figure 20. We observe that Prophet was better in all metrics as the XGBoost, generally, overfits this dataset.

## 4. Concluding Remarks and Further Steps

This work is about monitoring bioecological time series data originating from inside a beehive with a view to identify health hazards to the colony. A custom, low cost and power sufficient multisensory recorder gathers data from CO_2_ concentration (ppm), concentration of volatile compounds (ppb), temperature and humidity as well as counts of bees entering and exiting the hive. The platform also transmits exact sampling time, GPS coordinates and weight. These data constitute a multidimensional time series that can be analyzed by machine learning techniques to identify current trends in sensors’ values, predict future outcomes and regions of confidence around them but, most of all, identify atypical values that may relate to hazardous situations for the health of the beehive. In this work, we present our open, optimized in terms of electronic components and power, smart beehive, we comment on the multidimensional time series produced and we discuss ways machine learning techniques can be integrated to a decision support system that issues alert messages.

The following step, besides transmitting multidimensional data to be interpreted at the server, is to transmit direct estimation in the beehive of the health status (i.e., edge computing) that will allow beekeepers, researchers, and public authorities to be active participants in colony surveillance programs with increased accuracy and responsiveness. Our work, among many other, equally competitive approaches, [51,52] have a common goal: Unhealthy or threatened colonies to be remotely monitored for their health status, with greater precision, saving the cost of potential economic losses and preventing the loss of pollination services. Currently (as per 18/01/2023), the cost of the whole setup is EUR 200. In the near future, we intend to inflict stressful events to the beehive’s health and report how well the sensors have been able to pinpoint the event in the sensors’ readings. We will simulate health threats by intentionally stressing the beehive with various procedures (e.g., increase humidity, remove the queen, increase internally the levels of CO_2_) and we will observe the sensors’ readings to study to what extent the events can be detected, or their cumulative effect predicted.

## Figures and Tables

**Table 1:** Vibrational patterns of bees and their role.

Audio Signal	Freq. Bands (Hz)	Role	References
Whooping	300–450	begging call or stop signal	[18]
Queen piping	400–500	swarming, young queens to signal readiness for battle with the mature queen	[37]
Queen quacking	200–350	quacking follows tooting, confined queens responses	[40]
Tremble	300–450	foragers returning to the hive, stimulates additional bees to function as nectar receivers	[42]
Waggle	250–300	recruitment to feeding sites, inform nestmates about direction and distance to locations of attractive food	[43]
Tooting	200-350, 400-500	young queens to signal readiness for battle with the mature queen	[44]
Worker piping	100–250, 330–430	liftoff	[36,45]
Low signals	0–100	indicators of worker bees activity level	[46]

**Table 2:** Error metrics for the humidity variable.

	TRAIN	TEST
**MAPE**	5.99	5.01
**R^2^**	72.84	45.62

**Table 3:** Error metrics for the humidity variable.

	TRAIN	TEST
**MAPE**	1.67	8.36
**R^2^**	97.59	−17.49

## Data Availability

We share the research data and code to reproduce figures and experiments in this paper.

## Supplementary Materials

The full database is included in the associated csv file and the Python codes to re-produce all results of this work are parts of the publication. A video of the spectrogram of each 5 min recording is uploaded at https://zenodo.org/record/7455055#.Y59znXZBy3A.
